# Bacitracin enhances ceftriaxone susceptibility of the high-level ceftriaxone-resistant gonococcal FC428 clone

**DOI:** 10.1128/spectrum.02449-23

**Published:** 2023-11-20

**Authors:** Yuhua Gu, Shuaijie Song, Qingrui Zhu, Ruilin Jiao, Xu'ai Lin, Fan Yang, Stijn van der Veen

**Affiliations:** 1 Department of Microbiology, and Department of Dermatology of Sir Run Run Shaw Hospital, School of Medicine, Zhejiang University, Hangzhou, China; 2 College of Medicine, Shaoxing University, Shaoxing, China; 3 State Key Laboratory for Diagnosis and Treatment of Infectious Diseases, Collaborative Innovation Center for Diagnosis and Treatment of Infectious Diseases, The First Affiliated Hospital, School of Medicine, Zhejiang University, Hangzhou, China; 4 Zhejiang Provincial Key Laboratory for Microbial Biochemistry and Metabolic Engineering, Hangzhou, China; Yale University, New Haven, Connecticut, USA

**Keywords:** *Neisseria gonorrhoeae*, FC428, bacitracin, ceftriaxone, antimicrobial synergy, FICI, dual therapy

## Abstract

*Neisseria gonorrhoeae* is a multidrug-resistant bacterial pathogen for which ceftriaxone mono-antimicrobial therapy and ceftriaxone plus azithromycin dual-antimicrobial therapy are the only remaining effective therapies. However, ceftriaxone efficacy is threatened by the global dissemination of the high-level ceftriaxone-resistant FC428 clone, while azithromycin has in recent years been removed from the recommended dual therapy in some countries as a result of increasing (high-level) azithromycin resistance. Inclusion of an alternative second antimicrobial could be an effective strategy to protect ceftriaxone from (further) resistance development. In this study, we investigated gonococcal susceptibility to bacitracin and determined its synergistic anti-gonococcal activity with ceftriaxone. Gonococcal susceptibility to bacitracin was investigated for 449 contemporary clinical isolates using the agar dilution method. Bacitracin displayed consistent activity against the *N. gonorrhoeae* strain collection, with a minimum inhibitory concentration (MIC) range of 2–32 mg/L and a MIC_90_ of 32 mg/L. Furthermore, bacitracin was bactericidal in time-kill assays, particularly against the tested FC428-associated isolate, which showed 10^5^-fold inactivation after 8 hours of exposure to a 4× MIC dose. Importantly, bacitracin and ceftriaxone displayed synergistic activity, with a fractional inhibitory concentration index ≤0.5 for the majority of tested strains. Finally, spot assays showed strong synergistic activity against the FC428-associated isolates, with up to 200-fold reduced plating efficacy for bacitracin combined with ceftriaxone compared with ceftriaxone only. In conclusion, bacitracin shows synergistic anti-gonococcal activity with ceftriaxone and might therefore be an interesting candidate for inclusion in a dual-antimicrobial therapy with ceftriaxone.

**IMPORTANCE:**

Ceftriaxone-based antimicrobial therapies for gonorrhea are threatened by waning ceftriaxone susceptibility levels and the global dissemination of the high-level ceftriaxone-resistant gonococcal FC428 clone. Combination therapy can be an effective strategy to restrain the development of ceftriaxone resistance, and for that purpose, it is important to find an alternative antimicrobial to replace azithromycin, which has recently been removed in some countries from the recommended ceftriaxone plus azithromycin dual-antimicrobial therapy. Ideally, the second antimicrobial should display synergistic activity with ceftriaxone. We hypothesized that bacitracin might display synergistic activity with ceftriaxone because of their distinct mechanisms targeting bacterial cell wall synthesis. In this study, we showed that bacitracin indeed displays synergistic activity with ceftriaxone against *Neisseria gonorrhoeae*. Importantly, strains associated with the FC428 clone appeared to be particularly susceptible to the bacitracin plus ceftriaxone combination, which might therefore be an interesting dual therapy for further *in vivo* testing.

## INTRODUCTION

Gonorrhea is a prevalent sexually transmitted infection, with an estimated global incidence of 87 million new cases every year (1). There is currently no available vaccine that can provide enduring immunity (2, 3); therefore, antibiotics remain the only method for treatment for gonorrhea. However, *Neisseria gonorrhoeae* has developed resistance to all antibiotics that were previously recommended as first-line agents, including penicillin, tetracycline, ciprofloxacin, and azithromycin (4, 5). At present, ceftriaxone is the only remaining first-line mono-antimicrobial treatment option for gonorrhea, but the susceptibility of *N. gonorrhoeae* to ceftriaxone has decreased over the past decade (6 – 8). This is exemplified by sporadic reports of ceftriaxone treatment failure due to ceftriaxone-resistant isolates (9 – 11). Specifically, recent years have demonstrated global transmission of strains associated with the high-level ceftriaxone-resistant FC428 clone (minimum inhibitory concentration, MIC = 0.5–1 mg/L) (12 – 15), which carries semi-mosaic *penA* allele 60.001 that confers high-level ceftriaxone resistance due to its A311V and T483S polymorphisms (14, 16). Additionally, *penA* allele 60.001 also contains the polymorphisms I312M, F504L, N512Y, and G545S, which are related to reduced cephalosporin susceptibility (17, 18). High prevalence of strains associated with the FC428 clone, particularly in China and the Asia Pacific region (13, 15, 19 – 22), jeopardizes the effectiveness of ceftriaxone-based therapies for the treatment of gonorrhea.

Combination therapy has been acknowledged as an effective strategy to counteract the rapid increase in single-drug resistance (23), and there is a precedent for dual therapies in clinical settings for the treatment of gonorrhea. For instance, antimicrobial combinations of rifampicin (24) and erythromycin (25) were previously used for the treatment of gonorrhea, especially in instances of penicillin-resistant gonococcal infections (26 – 28). However, this dual therapy lost its efficacy with the development of rifampicin-resistant and erythromycin-resistant gonococcal isolates (29, 30). The combination of ceftriaxone and azithromycin is currently still recommended as first-line therapy in Europe, Australia, and Canada (31). However, with increasing incidences of azithromycin resistance in some regions and persistent clusters of strains displaying high-level azithromycin resistance (MIC ≥ 256 mg/L) (8, 32 – 34), updated treatment guidelines from the UK and USA no longer recommend inclusion of azithromycin in a dual therapy with ceftriaxone (35, 36). At present, ceftriaxone monotherapy constitutes the exclusive primary treatment option in clinical practice in most countries. However, over the past decade, recommended dosages have gradually increased to maintain effectiveness (31, 35 – 37), which is not a sustainable solution. Furthermore, there are presently no feasible alternative anti-gonococcal ceftriaxone-based combination therapies that are able to protect ceftriaxone against resistance development or enhance its effectiveness.

In our investigation, we discovered that bacitracin displayed synergism with ceftriaxone and enhanced ceftriaxone bactericidal activity and growth-limiting ability against *N. gonorrhoeae*. Bacitracin is a cyclic peptide antibiotic that binds C_55_-isoprenyl pyrophosphate (IPP), a lipid carrier that plays a crucial role in various biosynthetic processes, including bacterial glycoprotein biosynthesis (38). By inhibiting the dephosphorylation of IPP, bacitracin impedes the translocation of the bacterial peptidoglycan precursor N-acetylglucosamine/N-acetylmuramic acid disaccharide across the bacterial cell membrane, ultimately leading to the biosynthesis of a defective cell wall and bacterial lysis. In contrast, ceftriaxone targets bacterial transpeptidases (penicillin-binding proteins, PBPs), predominantly PBP2 in *N. gonorrhoeae*, which restricts cross-linking of the peptidoglycan chains and results in the biosynthesis of a defective cell wall. While both bacitracin and ceftriaxone interfere with bacterial cell wall synthesis, they target different stages of the process and may therefore positively affect each other’s activity. Unfortunately, oral bioavailability of bacitracin is limited (39, 40), while intramuscular injection might induce nephrotoxicity (41). However, bacitracin might be an interesting combination therapy with ceftriaxone for the treatment of gonococcal conjunctivitis or anorectal infections.

Breakpoints for gonococcal susceptibility to bacitracin are currently not defined, and previous research on gonococcal susceptibility to bacitracin is very limited. A study from the 1970s demonstrated by using *in vitro* peptidoglycan synthesis assays with ether-pretreated bacteria that bacitracin inhibited gonococcal peptidoglycan synthesis (42). Furthermore, it was recently shown that MIC of bacitracin against the gonococcal reference strain WHO Y was 4 mg/L (43). The current study aimed to assess antimicrobial synergy between bacitracin and ceftriaxone and further determine the susceptibility of *N. gonorrhoeae* to bacitracin more broadly using a large collection of contemporary multidrug-resistant clinical isolates, which included nine isolates associated with the FC428 clone.

## RESULTS

### Bacitracin susceptibility and cross-resistance analysis

**Fig 1:**
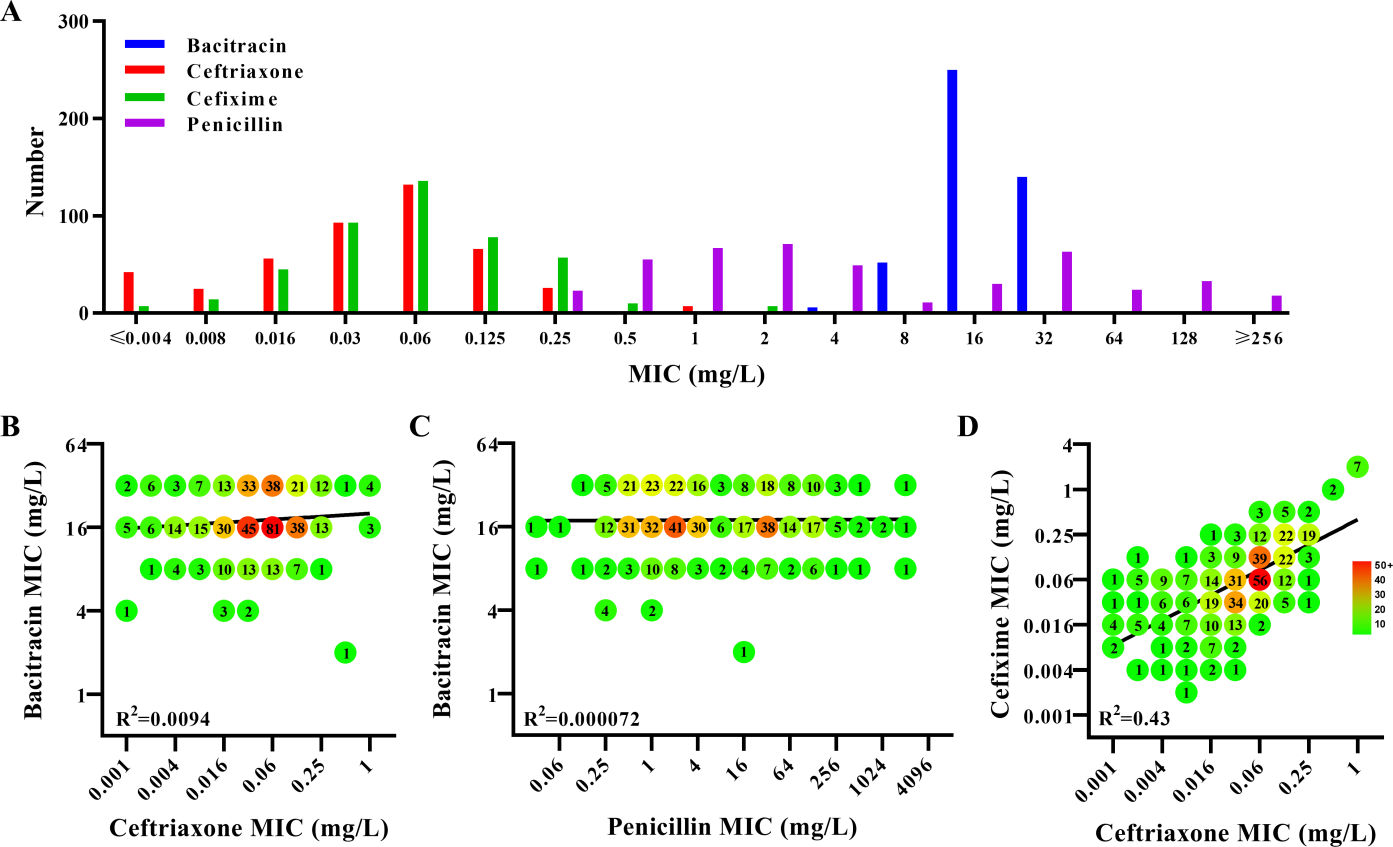
Gonococcal susceptibility to bacitracin and susceptibility correlation analysis with ceftriaxone and penicillin. (**A**) Distribution of bacitracin, ceftriaxone, cefixime, and penicillin MICs for 449 gonococcal isolates. (**B**) Correlation between MICs of bacitracin and ceftriaxone. (**C**) Correlation between MICs of bacitracin and penicillin. (**D**) Correlation between MICs of cefixime and ceftriaxone. Symbols in panels B–D represent one or multiple isolates indicated by values within the symbols and visualized by coloring. The regression line is shown.

The susceptibility of 449 clinical *N. gonorrhoeae* isolates (8, 15) to bacitracin and currently/previously recommended antimicrobials was tested by the agar dilution method (Table 1; Table S1). Overall, resistance against the previously/currently recommended antimicrobials ceftriaxone (7.8%; MIC > 0.125 mg/L), cefixime (17%; MIC > 0.125 mg/L), penicillin (67%; MIC > 1 mg/L), azithromycin (19%; MIC > 0.5 mg/L), tetracycline (77%; MIC > 1 mg/L), and ciprofloxacin (100%; MIC > 0.06 mg/L) was high. Furthermore, nine isolates showed high-level ceftriaxone resistance (MIC = 0.5–1 mg/L) and were associated with the FC428 clone. The MIC_50_ and MIC_90_ of bacitracin were 16 mg/L and 32 mg/L, respectively, with 98% of the strains displaying a MIC in the 8–32 mg/L range (Fig. 1A). The MIC correlation between bacitracin and ceftriaxone was poor, with a R = 0.097 (Fig. 1B). Similarly, a poor correlation was observed between MIC values for bacitracin and penicillin (R = 0.0085; Fig. 1C), while in contrast, the correlation between MIC values for ceftriaxone and cefixime was strong, with a R = 0.66 (Fig. 1D). Therefore, even though bacitracins like cephalosporins and penicillins target cell wall synthesis, the development of cross-resistance between bacitracin and ceftriaxone is unlikely.

**TABLE 1:** Susceptibility of 449 clinical *Neisseria gonorrhoeae* isolates to bacitracin and antimicrobials currently or previously used for gonorrhea treatment^*a*^

Antimicrobial	MIC range (mg/L)	MIC_50_ (mg/L)	MIC_90_ (mg/L)
Bacitracin	2–32	16	32
Ceftriaxone	0.001–1	0.06	0.125
Cefixime	0.002–2	0.06	0.25
Penicillin	0.03–2,048	4	128
Azithromycin	0.001–2,048	0.125	8
Tetracycline	0.008–128	2	64
Ciprofloxacin	0.03–128	16	32
Spectinomycin	1–64	32	32

^*a*^ MIC, minimum inhibitory concentration; MIC_50_, MIC that inhibits 50% of the isolates; MIC_90_, MIC that inhibits 90% of the isolates.

### Bacitracin bactericidal activity testing

**Fig 2:**
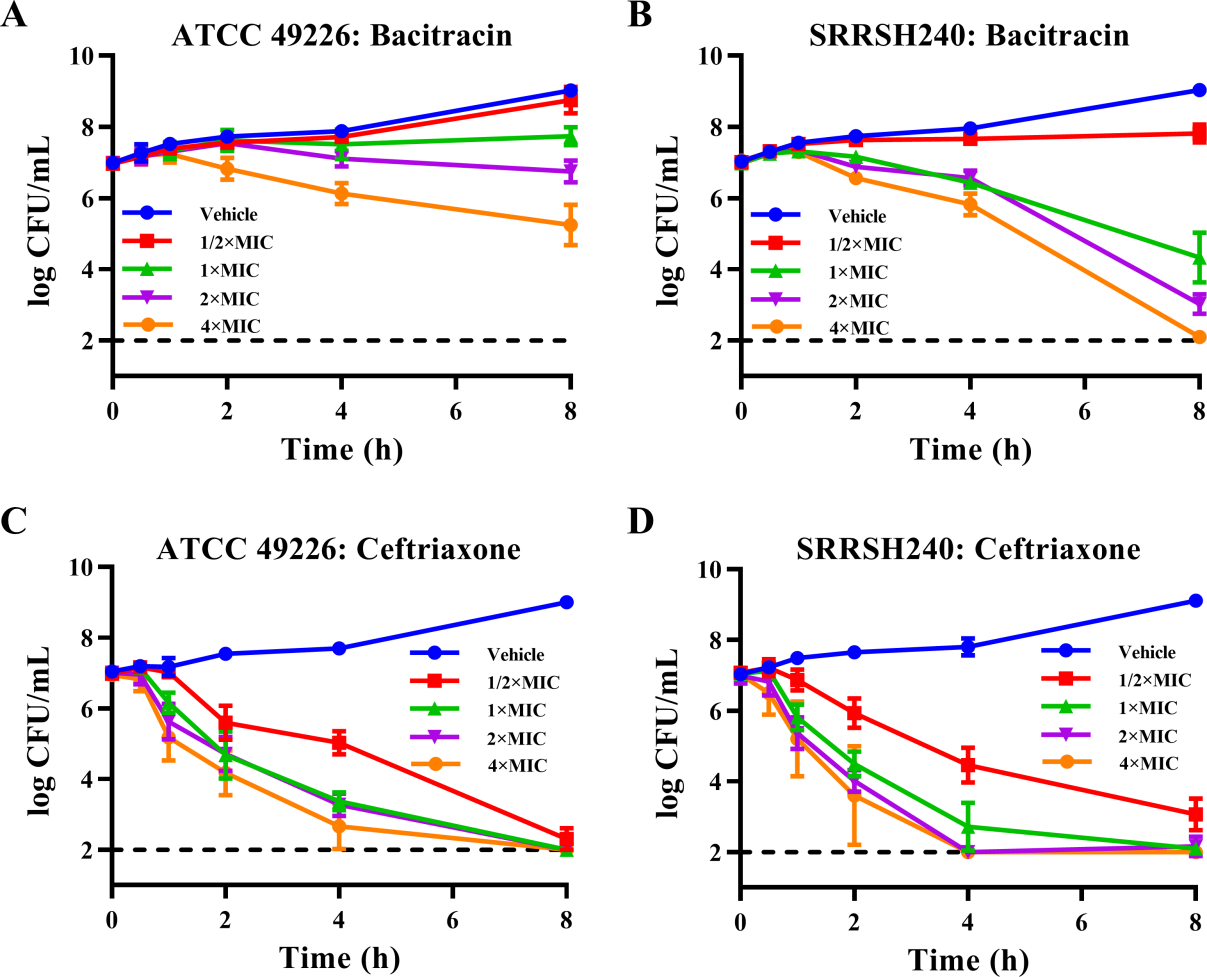
Bactericidal activity of bacitracin against *Neisseria gonorrhoeae* in time-kill assays. reference strain ATCC 49226 (**A, C**) and the FC428-associated isolate SRRSH240 (**B, D**) were exposed to bacitracin (**A, B**) or ceftriaxone (**C, D**) at 0.5×, 1×, 2×, or 4× the MIC or the vehicle control (GC broth only). Survival curves represent the mean and standard deviation of three biologically independent repeats. Bacitracin 1× MIC: 16 mg/L; ceftriaxone 1× MIC: 0.016 mg/L for ATCC 49226, 1 mg/L for SRRSH240.

The gonococcal reference strain ATCC 49226 and the high-level ceftriaxone-resistant FC428-associated isolate SRRSH240 (15) were investigated by time-kill assays to detect the bactericidal activity of bacitracin. Bacitracin showed moderate bactericidal activity for strain ATCC 49226, because after 8 hours of incubation with bacitracin at 4× MIC (64 mg/L), only 30-fold reduced CFU counts were observed (Fig. 2A). In contrast, FC428-associated isolate SRRSH240 showed 10^5^-fold inactivation after 8 hours of exposure to 4× MIC (64 mg/L), and even at 1× MIC, CFU counts were reduced by 200-fold (Fig. 2B). Therefore, it appears that bacitracin displays more potent bactericidal activity against the FC428-associated isolate compared with the ATCC 49226 reference strain. For reference, strains ATCC 49226 and SRRSH240 showed 10^5^-fold inactivation after incubation with ceftriaxone at 1× MIC for 8 hours (Fig. 2C and D), although 1× MIC ceftriaxone doses for ATCC 49226 (1× MIC = 0.016 mg/L) and SRRSH240 (1× MIC = 1 mg/L) differ markedly.

### Bacitracin synergy testing with ceftriaxone

**Fig 3:**
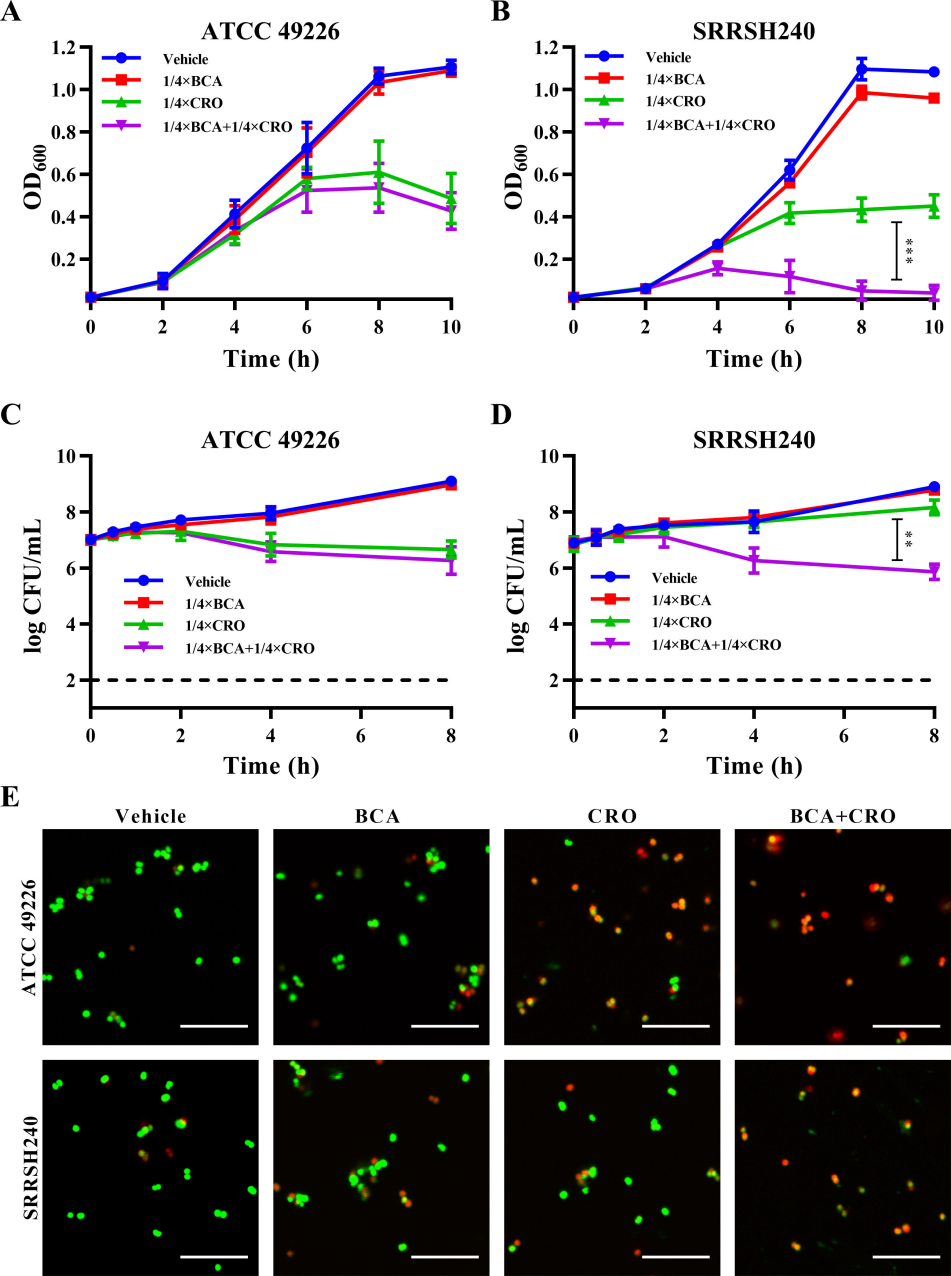
Synergistic activity between bacitracin and ceftriaxone in liquid culture. Reference strain ATCC 49226 (**A, C**) and the FC428-associated isolate SRRSH240 (**B, D**) were cultured in GC broth containing 1% Vitox and 0.25× the MIC of bacitracin (4 mg/L) and/or ceftriaxone (0.004 mg/L for ATCC 49226, 0.25 mg/L for SRRSH240). The vehicle control is GC broth. (**A, B**) Growth curves determined by absorbance measurements at OD_600_. (**C, D**) Viability curves determined by CFU determination. (**E**) Bacterial live/dead staining with SYTO9 (live; green/yellow) and propidium iodide (dead, red) after 4 hours of exposure to 0.25× the MIC of bacitracin and/or ceftriaxone. Scale bar: 10 µm. BCA: bacitracin; CRO: ceftriaxone. Significant differences between the curves were identified by analysis of variance (GraphPad Prism 8). **P < 0.01; ***P < 0.001.

**Fig 4:**
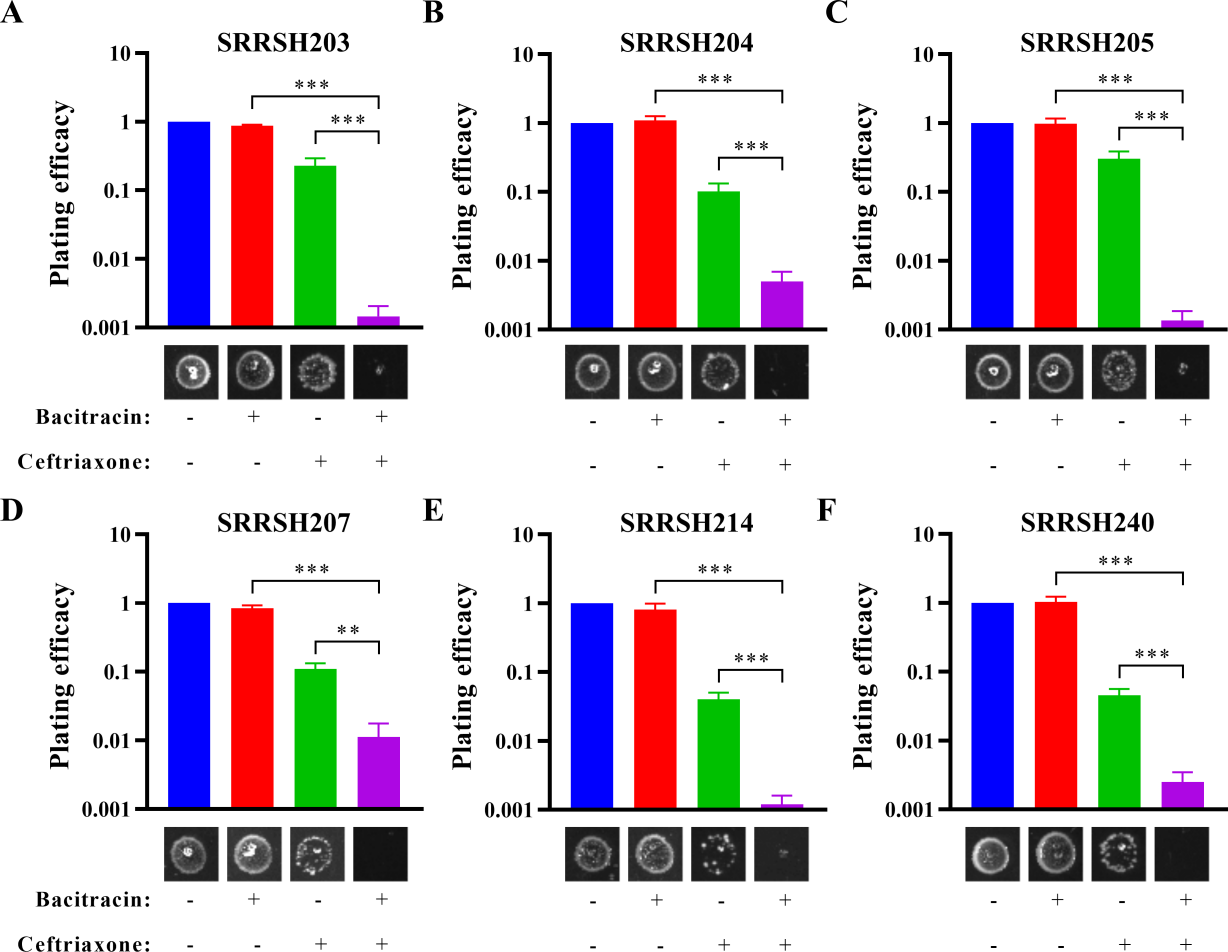
Synergistic activity between bacitracin and ceftriaxone against FC428-associated gonococcal isolates in spot assays. The graphs represent the plating efficacy of strains grown on agar plates containing bacitracin (12 mg/L) or ceftriaxone (0.45 mg/L) as single antimicrobial or combined antimicrobials relative to growth on control plates in the absence of antimicrobials. Representative images of bacterial growth are included. (**A**) Strain SRRSH203; (**B**) strain SRRSH204; (**C**) strain SRRSH205; (**D**) strain SRRSH207; (**E**) strain SRRSH214; (**F**) strain SRRSH240. Graphs represent the average and standard deviation of three biologically independent repeats. Significant differences were identified by Student’s two-tailed unpaired *t*-test (GraphPad Prism 8). **P < 0.01; ***P < 0.001.

Since ceftriaxone is the only remaining recommended first-line therapy for the treatment of gonococcal infections, inclusion of bacitracin in a dual therapy with ceftriaxone might be beneficial to enhance ceftriaxone activity and reduce further development of ceftriaxone resistance, particularly because bacitracin and ceftriaxone target different steps of bacterial cell wall biosynthesis. Therefore, the checkerboard method was used for antimicrobial synergy testing to determine the fractional inhibitory concentration index (FICI) for strain ATCC 49226 and 25 clinical isolates, which covered ceftriaxone MIC values in the range of 0.03–1 mg/L and included 6 FC428-associated isolates (Table 2). Of the 25 tested gonococcal clinical isolates, 15 isolates displayed synergistic activity between bacitracin and ceftriaxone (FICI ≤ 0.5), and the other 10 isolates displayed indifference (FICI > 0.5, <4). Therefore, bacitracin and ceftriaxone appear to display synergistic activity against the majority of clinical isolates, although for the FC428-associated isolates, synergistic activity was less strong with an average FICI of 0.79 (SEM = 0.077), as compared with an average FICI of 0.52 (SEM = 0.059) for the other isolates (*P* = 0.008, Mann-Whitney test). However, FICI-based synergy determination is limited in sensitivity because it is dependent on a growth-no-growth cut-off and therefore does not capture partial growth defects. Therefore, synergistic activity between bacitracin and ceftriaxone was determined in liquid culture using strains ATCC 49226 and SRRSH240 and antibiotics at 0.25× MIC. Both strains did not show a growth defect in the presence of bacitracin only compared with the vehicle control when growth was determined by absorbance (Fig. 3A and B) or CFU determination (Fig. 3C and D). In contrast, ceftriaxone only reduced the growth rate and maximum reached absorbance values for both strains when growth was determined by absorbance, while based on CFU determination, strain ATCC 49226 showed temporal increased CFU counts, which subsequently declined, and strain SRRSH240 only showed a limited growth defect at the latest time point. In contrast, when both antibiotics were combined at 0.25× MIC, strain SRRSH240 was effectively inhibited for growth, and bactericidal activity was observed from 2 hours onward. Bactericidal activity was confirmed by fluorescence microscopy using live/dead staining after 4 hours of exposure, which showed predominantly dead cells (red) for the bacitracin and ceftriaxone combination exposure of strain SRRSH240, while for the single antimicrobial or vehicle control conditions, abundant viable cells (green) were observed (Fig. 3E). In contrast, for strain ATCC 49226, bacitracin did not appear to enhance the bactericidal activity already observed for ceftriaxone at 0.25× MIC. Finally, synergistic activity between bacitracin and ceftriaxone was determined for six FC428-associated isolates by plating efficacy in spot assays using antibiotic concentrations close to the MIC (ceftriaxone MIC = 1 mg/L and bacitracin MIC = 16 mg/L for strains SRRSH203, SRRSH205, and SRRSH240 and 32 mg/L for strains SRRSH204, SRRSH207, and SRRSH214). The combination of bacitracin and ceftriaxone reduced the plating efficacy by 10- to 200-fold compared with plating efficacy for ceftriaxone only (Fig. 4), highlighting that bacitracin enhances the antimicrobial activity of ceftriaxone for all FC428-associated strains.

**TABLE 2:** *Neisseria gonorrhoeae* FICI for bacitracin combined with ceftriaxone^*a*^

Isolate ID	NG-MAST	MIC (mg/L)		FICI Bacitracin + Ceftriaxone
		Bacitracin	Ceftriaxone	
SRRSH 190	ST17900	16	0.03	0.56
SRRSH 192	ST5061	32	0.03	0.38
SRRSH 225	ST21359	32	0.03	1.25
SRRSH 185	ST21327	32	0.06	0.50
SRRSH 186	ST21328	16	0.06	0.53
SRRSH 201	ST21357	16	0.06	0.50
SRRSH 210	ST13542	32	0.06	0.50
SRRSH 215	ST3741	32	0.06	0.50
SRRSH 182	ST21325	32	0.125	1.13
SRRSH 184	ST21326	32	0.125	0.31
SRRSH 188	ST21349	32	0.125	0.38
SRRSH 202	ST21330	16	0.125	0.50
SRRSH 208	ST21332	16	0.125	0.31
SRRSH 211	ST21358	16	0.125	0.50
SRRSH 189	ST21350	16	0.25	0.38
SRRSH 193	ST21329	32	0.25	0.63
SRRSH 194	ST21345	16	0.25	0.31
SRRSH 199	ST21356	32	0.25	0.31
SRRSH 200	ST5061	16	0.25	0.31
SRRSH 203	ST19972	16	1	1.00
SRRSH 204	ST19972	32	1	0.75
SRRSH 205	ST19972	16	1	1.00
SRRSH 207	ST19973	32	1	0.50
SRRSH 214	ST19972	32	1	0.75
SRRSH 240	ST19973	16	1	0.75

^*a*^ FICI, fractional inhibitory concentration index; NG-MAST, *N. gonorrhoeae* multiantigen sequence typing; MIC, minimum inhibitory concentration.

## DISCUSSION

*N. gonorrhoeae* has developed resistance to all antimicrobial therapies that are either currently in use or have previously been clinically recommended. Ceftriaxone is currently the only remaining mono-antimicrobial therapy that can securely cure gonorrhoea from all common infection sites (44, 45). However, in some regions, ceftriaxone susceptibility levels have gradually decreased over the past decade (6 – 8, 46, 47), while in regions where treatment commonly consists of ceftriaxone plus azithromycin dual therapy, the decrease in susceptibility levels appears to be less pronounced (23). Unfortunately, continuous high incidences of azithromycin resistance and outbreaks of strains displaying high-level azithromycin resistance have led to the exclusion of azithromycin from dual therapy in some countries (35, 36). To delay further emergence and spread of ceftriaxone resistance in *N. gonorrhoeae*, the inclusion of an alternative antimicrobial in a dual therapy with ceftriaxone could be beneficial.

The current study revealed that bacitracin not only possesses consistent antimicrobial activity against a large collection of clinical *N. gonorrhoeae* isolates but also enhances the anti-gonococcal activity of ceftriaxone. Bacitracin is a cyclic polypeptide antibiotic composed of 12 amino acids that inhibits dephosphorylation of the lipid carrier involved in the translocation of cell wall precursors across the cell membrane, thereby inhibiting cell wall synthesis (38, 48). Ceftriaxone also targets cell wall synthesis but instead inactivates transpeptidases involved in the cross-linking of peptidoglycan chains. Based on their distinct mechanisms of action against bacterial cell wall synthesis, we hypothesized that the combined use of bacitracin and ceftriaxone might enhance their anti-gonococcal effects. Indeed, our FICI analysis indicated that bacitracin and ceftriaxone display synergistic activity against most of the tested clinical isolates. Furthermore, synergistic activity was particularly evident for FC428-associated strains in spot assays. While bacitracin is generally considered a narrow-spectrum antimicrobial that is mostly limited to Gram-positive bacteria (49 – 51), some activity against *N. gonorrhoeae* has previously been demonstrated (42, 43), including a recent investigation that reported a MIC of 4 mg/L against the gonococcal reference strain WHO Y (43). Our study assessed bacitracin susceptibility for 449 recent clinical gonococcal isolates and revealed that all isolates displayed MICs within the 2–32 mg/L range. However, since there are no established breakpoints for gonococcal susceptibility to bacitracin, it is currently not possible to interpret the observed bacitracin MIC range in relation to clinical efficacy.

Bacitracin is commonly prescribed in combination with polymyxin B and neomycin for ophthalmic or topical use or to treat skin infections (51, 52). These applications may come in the form of eye ointments, salves, or sprays and are used for external treatments. When applied as an ointment for the treatment of skin infections, including wound infections and cellulitis, bacitracin exhibits no observable toxicity (53, 54). Bacitracin has also been used to prevent visual impairment in neonates, specifically in cases of neonatal conjunctivitis caused by gonococcal infections (52, 55). If left untreated or inadequately treated, this infection can result in corneal perforation and loss of vision within 24 hours (56). Drug resistance also poses a challenge for the treatment of gonococcal conjunctivitis (57), and the combination therapy of ophthalmic bacitracin and injectable ceftriaxone might therefore be an interesting alternative therapeutic option. Bacitracin can also be administered through oral, intramuscular, and intrathecal routes; however, oral administration of bacitracin is not commonly recommended due to its low oral bioavailability (39, 40). Interestingly, *N. gonorrhoeae* is a common cause of anorectal infections in the population of men who have sex with men (MSM), and oral bacitracin has successfully been used to clear vancomycin-resistant *Enterococcus faecium* infections from the gastrointestinal tract (58 – 61). Therefore, oral bacitracin might be an interesting alternative combination therapy with injectable ceftriaxone for the treatment of anorectal gonococcal infections.

Intramuscular injections of bacitracin may induce nephrotoxicity, leading to renal failure and thrombophlebitis (41). Until recently, bacitracin was U.S. Food and Drug Administration (FDA)-approved for intramuscular use in infants to treat pneumonia and empyema caused by staphylococci (62); however, given its nephrotoxicity and the availability of alternative treatments, the FDA requested the removal of bacitracin for injection from the market in 2020. Regardless, it is tempting to speculate whether the required injectable bacitracin doses could be sufficiently lowered to avoid nephrotoxicity when used in combination therapy with ceftriaxone. A beneficial effect of the bacitracin and ceftriaxone combination was already observed at 0.25× MIC, which combined with a MIC_90_ of 32 mg/L for *N. gonorrhoeae* indicates that the minimum bacitracin dose should reach a C_max_ ≥ 8 mg/L. Although a specific bacitracin dose-effect relationship with nephrotoxicity is not well established, the reported intravenous LD_50_ of bacitracin in mice is 360 mg/kg, and serum concentrations up to 2 mg/L can be obtained with intramuscular doses as low as 6 mg/kg (62). Furthermore, bacitracin is mostly secreted through the urine, which might be beneficial for obtaining elevated concentrations to target urogenital *N. gonorrhoeae* infections. For ceftriaxone, intravenous doses of 0.5–2 g provide plasma C_max_ values of 80–250 mg/L (63 – 65), while an intramuscular 0.5 g ceftriaxone dose results in plasma C_max_ values around 40–45 mg/L (66). Plasma ceftriaxone levels above 1 mg/L, which is commonly the MIC for FC428-associated strains, can be maintained for over 24 hours with these intravenous or intramuscular doses. Similar to bacitracin, ceftriaxone also shows urinal accumulation, which might therefore be beneficial for a combined antimicrobial therapy for urogenital gonorrhea. However, further research on bacitracin pharmacokinetics and nephrotoxicity is required to determine whether a combined therapy of injectable bacitracin plus ceftriaxone is feasible.

In conclusion, this study assessed the antimicrobial activity of bacitracin against 449 clinical multidrug-resistant isolates. Our findings indicate that bacitracin is active against all tested isolates, including strains associated with the high-level ceftriaxone-resistant FC428 clone. Bacitracin is a bactericidal antimicrobial for *N. gonorrhoeae* and furthermore displayed synergistic activity with ceftriaxone, which was particularly evident in spot assays for strains associated with the FC428 clone. Bacitracin might therefore be an interesting antimicrobial for inclusion in future anti-gonococcal dual therapies with ceftriaxone for the treatment of gonococcal conjunctivitis or anorectal infections, although further *in vivo* studies are required to establish the *in vivo* activity and efficacy of a possible bacitracin plus ceftriaxone dual-antimicrobial therapy.

## MATERIALS AND METHODS

### Determination of antimicrobial minimum inhibitory concentrations

Susceptibility of *N. gonorrhoeae* to bacitracin (Aladdin), ceftriaxone (Meilunbio), and reference antimicrobials was determined by the agar dilution method (67) for 449 clinical isolates covering the period 2015–2017 (8) and 2019 (15). Bacteria were spotted at approximately 10^4^ CFU on GC agar (Oxoid) plates containing 1% (vol/vol) Vitox (Oxoid) and antimicrobials at the required concentrations. The international reference strain for antimicrobial susceptibility testing, ATCC 49226, was included for quality control. Agar plates were incubated for 24 hours at 37°C and 5% CO_2_ before growth was determined. The MIC was defined as the lowest antimicrobial concentration at which no growth was observed. A linear regression analysis of log-transformed MIC values was performed to determine the correlation in MIC values between bacitracin and ceftriaxone or penicillin, with ceftriaxone and cefixime as positive references.

### Antimicrobial synergy testing by the chequerboard method

The chequerboard agar dilution method (68) was used to determine synergistic activity between bacitracin and ceftriaxone for reference strain ATCC 49266 and 25 clinical isolates covering a range of ceftriaxone MIC values. The FICI was determined based on MIC values for bacitracin and ceftriaxone as single antimicrobials (MIC_BAC/CRO_^single^) and when combined (MIC_BAC/CRO_^combined^) using the formula:


$$FICI=\frac{MIC{{}_{\text{BAC}}}^{combined}}{MIC{{}_{\text{BAC}}}^{\text{single}}}+\frac{MIC{{}_{\text{CRO}}}^{\text{combined}}}{MIC{{}_{\text{CRO}}}^{\text{single}}}$$

Antimicrobial synergy was defined by a FICI ≤ 0.5 and indifference by a FICI > 1 and ≤4.

### Liquid culture and time-kill assays

*N. gonorrhoeae* reference strain ATCC 49226 and the high-level ceftriaxone-resistant FC428-associated isolate SRRSH240 were revived overnight on GC agar containing 1% Vitox. Subsequently, bacteria were suspended in 12 mL GC broth [15 g/L peptone (Oxoid), 1 g/L soluble starch (Vetec), 5 g/L sodium chloride (Aladdin), 4 g/L dipotassium hydrogen phosphate (Vetec), 1 g/L potassium dihydrogen phosphate (Vetec)] containing 1% Vitox at a concentration of approximately 10^7^ CFU/mL or an OD_600_ = 0.02. For single antimicrobial time-kill assays, bacitracin or ceftriaxone was added at 0.5×, 1×, 2×, or 4× the MIC and GC broth only was the vehicle control. Cultures were incubated at 37°C and 200 rpm, and at intervals, samples were taken to determine survival on GC agar containing 1% Vitox. For liquid culture assays combining antimicrobials, bacitracin, or ceftriaxone were added as a single antimicrobial or in combination at 0.25× the MIC, depending on which concentration provided the most pronounced results, with GC broth only being the vehicle control. Cultures were incubated at 37°C and 200 rpm, and at intervals, samples were taken to determine the absorbance (OD_600_) or survival on GC agar containing 1% Vitox. Bacterial viability of cultures after single antimicrobial and combination exposure for 4 hours at 0.25× MIC was confirmed by confocal microscopy analysis after live/dead staining. Samples were washed with phosphate buffered saline (PBS) and incubated with SYTO 9 (Thermo; 1.67 µM) and propidium iodide (Thermo; 10 µM) for 15 min at room temperature. Bacteria were subsequently washed with PBS, and fluorescence was visualized with an Olympus FV1000 confocal microscope.

### Plating efficacy in a spot assay

The six FC428-associated high-level ceftriaxone-resistant SRRSH203, SRRSH204, SRRSH205, SRRSH207, SRRSH214, and SRRSH240 (15) were revived overnight on GC agar containing 1% Vitox. Bacterial suspensions were prepared at an OD_600_ = 1, and 10-fold dilution series were spotted on GC agar plates containing 1% Vitox and bacitracin (12 mg/L) or ceftriaxone (0.45 mg/L) as single antimicrobials or in combination with GC broth only as vehicle control. Agar plates were incubated for 24 hours at 37°C with 5% CO_2_, and single colonies were determined where possible. Plating efficacy was determined based on relative differences compared with the vehicle control.

## SUPPLEMENTAL MATERIAL

Table S1 spectrum.02449-23-s0001.xlsx Genotype and antimicrobial susceptibility of gonococcal strains used in the current study.

ASM does not own the copyrights to Supplemental Material that may be linked to, or accessed through, an article. The authors have granted ASM a non-exclusive, world-wide license to publish the Supplemental Material files. Please contact the corresponding author directly for reuse.
